# Supplementary material for: Molecular basis of retinal remodeling in a zebrafish model of retinitis pigmentosa
Source: Cell Mol Life Sci. 2023 Nov 18;80(12):362. doi: 10.1007/s00018-023-05021-1 (PMC10657301; doi:10.1007/s00018-023-05021-1)
Supplement: Supplementary file 1 — Supplementary file1 (DOCX 1697 KB) [file 18_2023_5021_MOESM1_ESM.docx]

**Molecular basis of retinal remodeling in a Zebrafish model of Retinitis Pigmentosa**

Cell and Molecular Life Sciences

Abirami Santhanam^¶*^, Eyad Shihabeddin^¶^, Haichao Wei, Jiaqian Wu, John O’Brien^*^

¶ Contributed equally

* Corresponding authors:

Abirami Santhanam: [abirami.santhanam@bcm.edu](mailto:abirami.santhanam@bcm.edu)

Human Genome Sequencing Center, Baylor College of Medicine, Houston, Texas, USA, 77030

John O’Brien: [jobrien3@Central.UH.EDU](mailto:jobrien3@Central.UH.EDU)

University of Houston College of Optometry, Houston, Texas, USA, 77204

**Supplementary Figures 1 – 5**


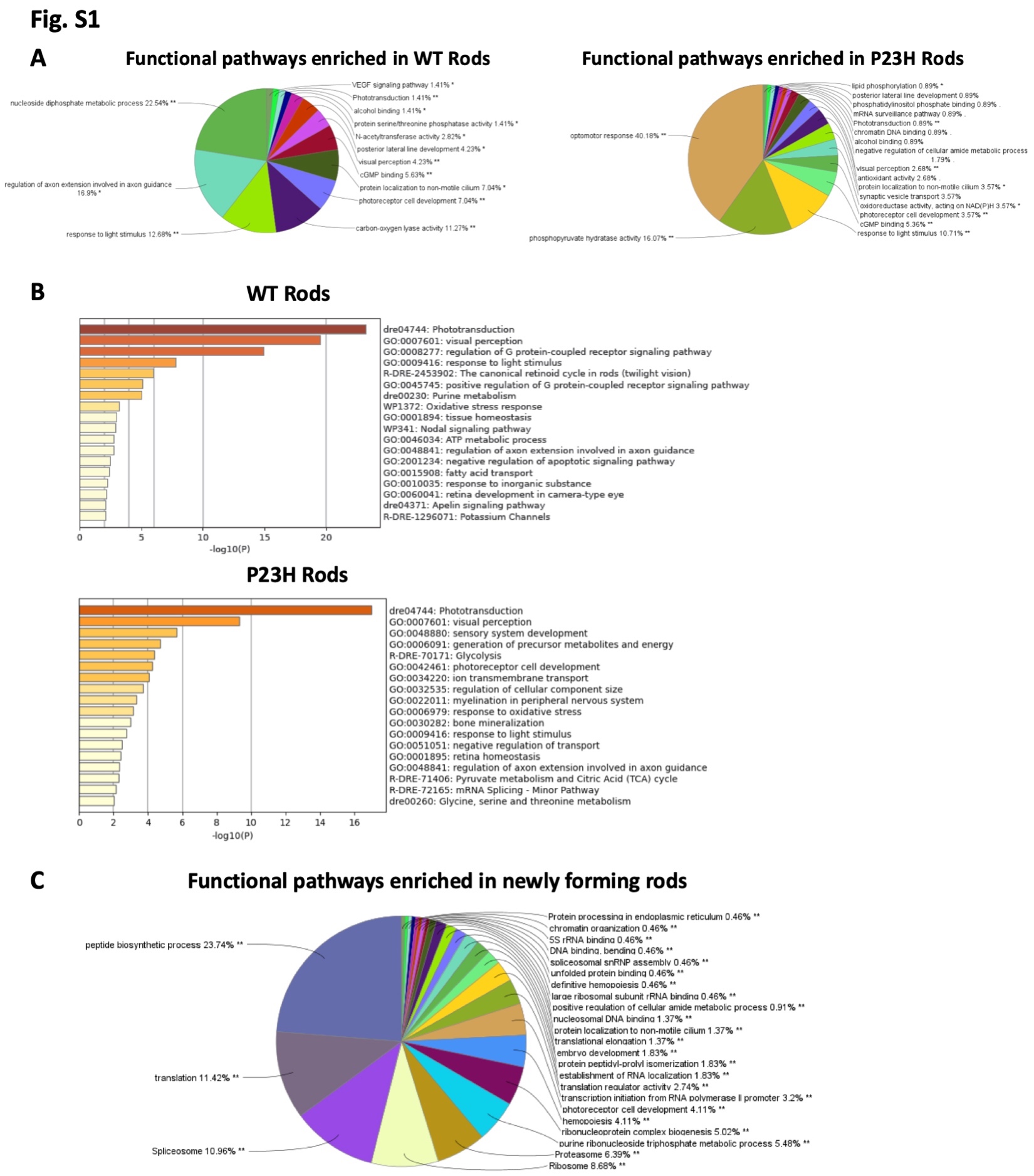


**Fig S1.** **Changes in rod functional pathways between WT and P23H.** **A**) Functional pathways enriched in the WT and P23H rods are revealed by Cytoscape analysis as described in the methods. **B**) The most prominent functional pathways present in the WT and P23H rods revealed by Metascape analysis. **C**) Functional pathways specifically enriched in the newly formed rods revealed by Cytoscape analysis.


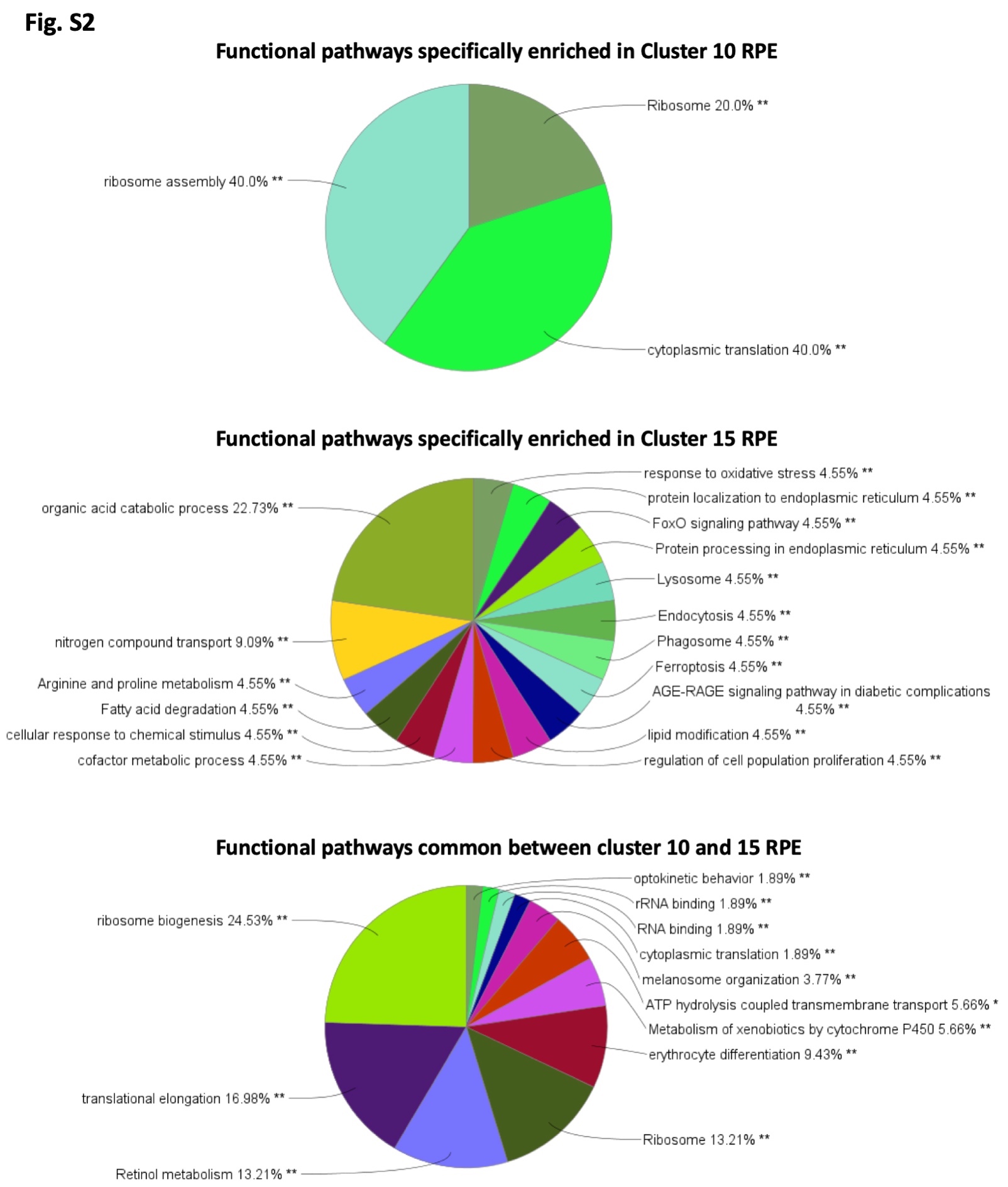


**Figure S2.** **Functional pathway analysis of RPE cells by Cytoscape.** Functional pathways specifically enriched in RPE clusters 10 and 15, and functional pathways shared by clusters 10 and 15


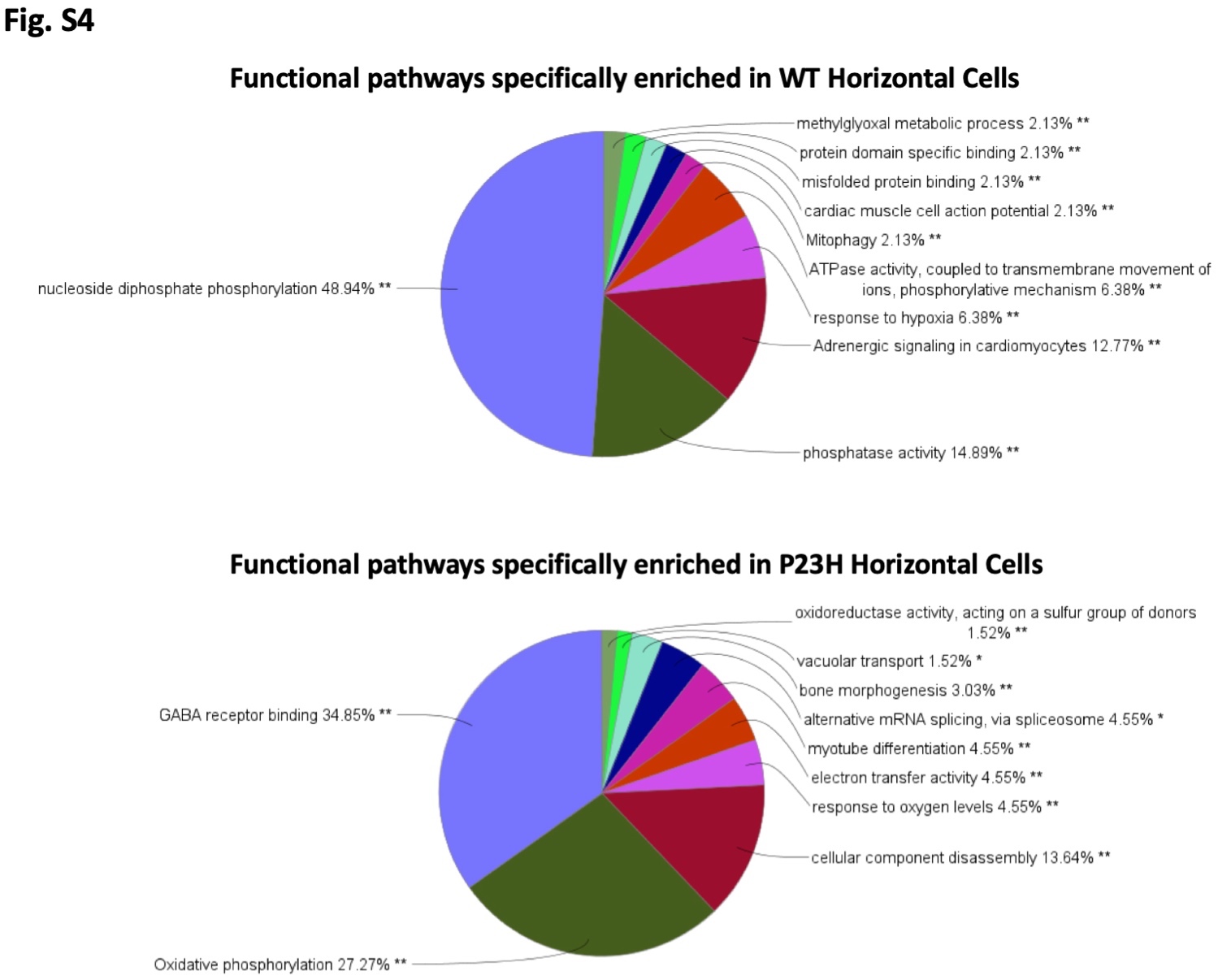


**Figure S3.** **Functional pathway analysis of horizontal cells by Cytoscape.** Functional pathways specifically enriched in the WT and P23H horizontal cells.


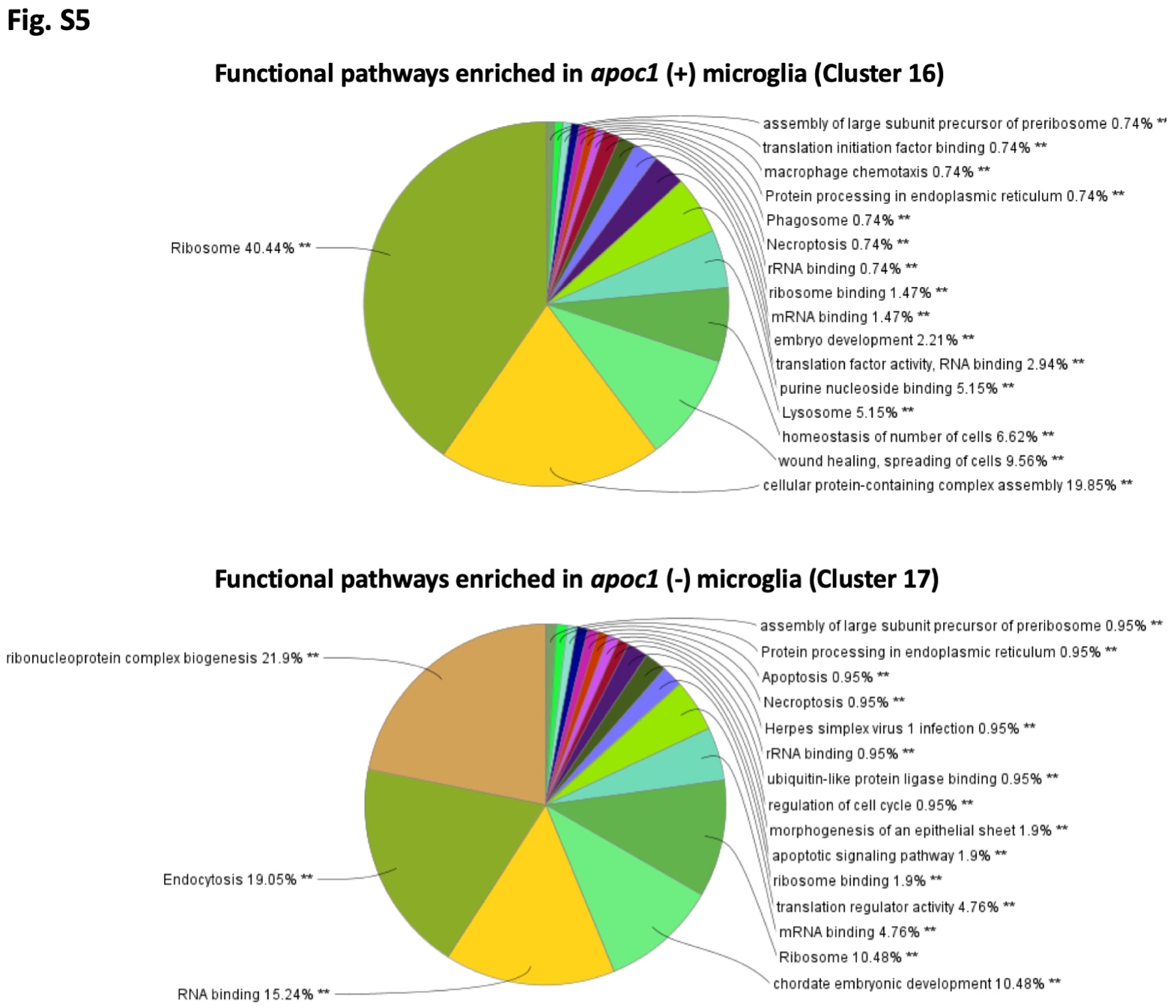


**Figure S4.** **Functional pathway analysis of** **microglia/macrophages by Cytoscape.** Functional pathways specifically enriched in the *apoc1*(+) and *apoc1*(-) microglia/macrophages.


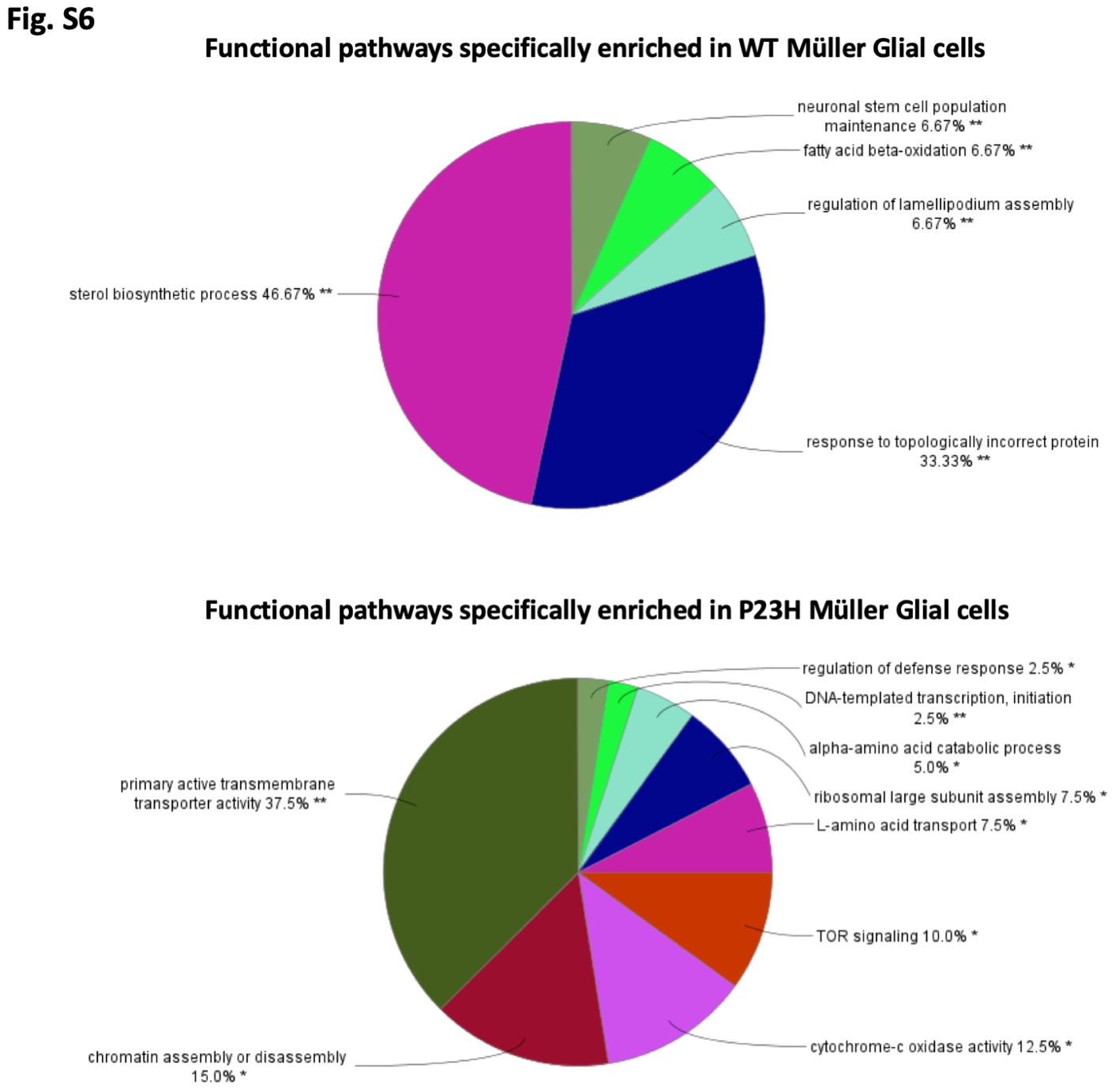


**Figure S5.** **Functional pathway analysis of Müller glial cells by Cytoscape.** Functional pathways specifically enriched in the WT and P23H Müller glial cells.
