# Supplementary material for: Molecular basis of retinal remodeling in a zebrafish model of retinitis pigmentosa
Source: Cell Mol Life Sci. 2023 Nov 18;80(12):362. doi: 10.1007/s00018-023-05021-1 (PMC10657301; doi:10.1007/s00018-023-05021-1)
Supplement: Supplementary file 2 — Supplementary file2 (DOCX 101 KB) [file 18_2023_5021_MOESM2_ESM.docx]

**Molecular basis of retinal remodeling in a Zebrafish model of Retinitis Pigmentosa**

Cell and Molecular Life Sciences

Abirami Santhanam^¶*^, Eyad Shihabeddin^¶^, Haichao Wei, Jiaqian Wu, John O’Brien^*^

¶ Contributed equally

* Corresponding authors:

Abirami Santhanam: [abirami.santhanam@bcm.edu](mailto:abirami.santhanam@bcm.edu)

Human Genome Sequencing Center, Baylor College of Medicine, Houston, Texas, USA, 77030

John O’Brien: [jobrien3@Central.UH.EDU](mailto:jobrien3@Central.UH.EDU)

University of Houston College of Optometry, Houston, Texas, USA, 77204

**Supplementary Tables 1 – 5**

Table S1. **Marker genes used to identify cell types**

| **Gene markers** | **Cell Type** | **Gene ID** | **Source** |
| --- | --- | --- | --- |
| *cabp1a* | Amacrine Cells | 449789 | Characterization of the Calcium Binding Protein Family in Zebrafish [1] |
| *tfap2a* | Amacrine Cells | 140618 | Tfap2a and 2b act downstream of Ptf1a to promote amacrine cell differentiation during retinogenesis [2] |
| *syt2a* | Amacrine Cells | 567877 | Localization of the Calcium-binding Protein Secretagogin in Cone Bipolar Cells of the Mammalian Retina [3] |
| *chata, gad2* | Amacrine Cells | 100170938, 550403 | Molecular identification of sixty-three amacrine cell types completes a mouse retinal cell atlas [4] |
| *cabp2a* | Bipolar Cells | 572226 | Characterization of the Calcium Binding Protein Family in Zebrafish [1] |
| *prkca* | Bipolar Cells | 497384 | A comparative analysis of rod bipolar cell transcriptomes identifies novel genes implicated in night vision [5] |
| *vsx1* | Bipolar Cells | 30598 | Vsx2 in the zebrafish retina: restricted lineages through derepression [6] |
| *rs1a* | Bipolar Cells | 445044 | Identification of molecular markers of bipolar cells in the murine retina [7] |
| *bhlhe23* | Bipolar Cells | 559796 | A comparative analysis of rod bipolar cell transcriptomes identifies novel genes implicated in night vision [5] |
| *gnat1* | Rods | 140428 | Transcripts within rod photoreceptors of Zebrafish Retina [8] |
| *rom1a, rom1b* | Rods | 393767, 393989 | Rom-1 is required for rod photoreceptor viability and the regulation of disk morphogenesis [9] |
| *saga, sagb* | Rods | 792319, 619268 | Transcripts within rod photoreceptors of Zebrafish Retina [8] |
| *gnat2* | Cones | 140429 | Transducin Duplicates in the Zebrafish Retina and Pineal Complex: Differential Specialisation after the Teleost Tetraploidisation [10] |
| *pde6c* | Cones | 393845 | A Mutation in the Cone-Specific pde6 Gene Causes Rapid Cone Photoreceptor Degeneration in Zebrafish [11] |
| *clul1* | Cones | 559018 | Comparative Analysis and Expression of CLUL1, a Cone Photoreceptor-Specific Gene [12] |
| *cx52.6* | Horizontal Cells | 404207 | Specific connectivity between photoreceptors and horizontal cells in the zebrafish retina [13] |
| *cx55.5* | Horizontal Cells | 573483 | Specific connectivity between photoreceptors and horizontal cells in the zebrafish retina [13] |
| *cx52.9* | Horizontal Cells | 404625 | Specific connectivity between photoreceptors and horizontal cells in the zebrafish retina [13] |
| *her4.3* | Retinal Progenitor Cells | 792198 | Tracking the fate of her4 expressing cells in the regenerating retina using her4:Kaede zebrafish [14] |
| *insm1a* | Retinal Progenitor Cells | 402941 | Insm1a-mediated gene repression is essential for the formation and differentiation of Müller glia-derived progenitors in the injured retina [15] |
| *her15.1* | Retinal Progenitor Cells | 100534909 | The transcription factor hairy/E(spl)-related 2 induces proliferation of neural progenitors and regulates neurogenesis and gliogenesis [16] |
| *rpe65a* | Retinal Pigment Epithelial Cells | 393724 | Expression profiling of the RPE in zebrafish smarca4 mutant revealed altered signals that potentially affect RPE and retinal differentiation [17] |
| *dct* | Retinal Pigment Epithelial Cells | 58074 | Expression profiling of the RPE in zebrafish smarca4 mutant revealed altered signals that potentially affect RPE and retinal differentiation [17] |
| *pmela, pmelb* | Retinal Pigment Epithelial Cells | 321239, 562810 | Identification of cell surface markers and establishment of monolayer differentiation to retinal pigment epithelial cells [18] |
| *rbpms2a, rbpms2b* | Retinal Ganglion Cell | 436682 | The RNA binding protein RBPMS is a selective marker of ganglion cells in the mammalian retina [19] |
| *pou4f1* | Retinal Ganglion Cell | 58057 | Genetic interplay between transcription factor Pou4f1/Brn3a and neurotrophin receptor Ret in retinal ganglion cell type specification [20] |
| *isl2b* | Retinal Ganglion Cell | 30151 | Molecular classification of zebrafish retinal ganglion cells links genes to cell types to behavior [21] |
| *plp1b* | Oligodendrocytes | 368234 | Identification of genes expressed by zebrafish oligodendrocytes using a differential microarray screen [22] |
| *cd59* | Oligodendrocytes | 567192 | Cd59 and inflammation regulate Schwann cell development [23] |
| *cd9b* | Oligodendrocytes | 406737 | Expression and distribution of CD9 in myelin of the central and peripheral nervous systems [24] |
| *gfap* | Müller Glial Cells | 30646 | Zebrafish Lbh-like Is Required for Otx2-mediated Photoreceptor Differentiation [25] |
| *cahz* | Müller Glial Cells | 30331 | Characterization of Müller glia and neuronal progenitors during adult zebrafish retinal regeneration [26] |
| *ptgdsb.1* | Müller Glial Cells | 336492 | Rapid, Dynamic Activation of Müller Glial Stem Cell Responses in Zebrafish [27] |
| *cd74a* | Microglia | 58113 | Regeneration associated transcriptional signature of retinal microglia and macrophages [28] |
| *cd74b* | Microglia | 30645 | Regeneration associated transcriptional signature of retinal microglia and macrophages [28] |
| *apoc1* | Microglia | 570638 | Regeneration associated transcriptional signature of retinal microglia and macrophages [28] |

Table S2. **Top 10 genes from each Wild Type cluster**

| **gene** | **p_val** | **avg_log2FC** | **pct.1** | **pct.2** | **p_val_adj** | **cluster** |
| --- | --- | --- | --- | --- | --- | --- |
| *cabp5a* | 0 | 3.930352 | 0.958 | 0.455 | 0 | 0 |
| *cabp2a* | 0 | 3.328842 | 0.795 | 0.156 | 0 | 0 |
| *rs1a* | 0 | 2.985809 | 0.652 | 0.189 | 0 | 0 |
| *lrit1a* | 0 | 2.933814 | 0.634 | 0.111 | 0 | 0 |
| *si:ch73-256g18.2* | 0 | 2.905666 | 0.837 | 0.17 | 0 | 0 |
| *vamp1* | 0 | 2.784025 | 0.894 | 0.229 | 0 | 0 |
| *efna1b* | 0 | 2.776288 | 0.84 | 0.328 | 0 | 0 |
| *waslb* | 0 | 2.503441 | 0.643 | 0.178 | 0 | 0 |
| *vsx1* | 0 | 2.491107 | 0.497 | 0.082 | 0 | 0 |
| *nrn1lb* | 0 | 2.338138 | 0.524 | 0.088 | 0 | 0 |
| *opn1lw2* | 1.22E-66 | 4.441128 | 0.286 | 0.11 | 2.3E-62 | 1 |
| *opn1lw1-1* | 3.9E-186 | 4.40282 | 0.447 | 0.133 | 7.4E-182 | 1 |
| *opn1lw2-1* | 4.9E-113 | 4.352769 | 0.558 | 0.289 | 9.2E-109 | 1 |
| *opn1mw2* | 2.87E-37 | 3.886074 | 0.265 | 0.135 | 5.42E-33 | 1 |
| *clul1* | 0 | 3.494121 | 0.938 | 0.282 | 0 | 1 |
| *hexb* | 0 | 3.365444 | 0.806 | 0.179 | 0 | 1 |
| *LOC100334711* | 0 | 3.099652 | 0.768 | 0.112 | 0 | 1 |
| *prph2a* | 0 | 3.064058 | 0.892 | 0.204 | 0 | 1 |
| *arr3a-1* | 2.3E-269 | 2.866967 | 0.868 | 0.555 | 4.3E-265 | 1 |
| *htra1b* | 0 | 2.860907 | 0.823 | 0.13 | 0 | 1 |
| *gad2* | 0 | 3.287965 | 0.926 | 0.128 | 0 | 2 |
| *LOC557301* | 1.2E-120 | 3.127811 | 0.335 | 0.093 | 2.3E-116 | 2 |
| *crhbp* | 1.27E-60 | 2.722586 | 0.254 | 0.09 | 2.4E-56 | 2 |
| *scg2b* | 0 | 2.641145 | 0.676 | 0.108 | 0 | 2 |
| *egr4* | 5.3E-213 | 2.624786 | 0.689 | 0.247 | 1E-208 | 2 |
| *slc6a1b* | 0 | 2.58459 | 0.855 | 0.08 | 0 | 2 |
| *snap25a* | 0 | 2.576376 | 0.957 | 0.329 | 0 | 2 |
| *slc6a1a* | 0 | 2.56403 | 0.865 | 0.067 | 0 | 2 |
| *vamp2* | 0 | 2.453991 | 0.959 | 0.294 | 0 | 2 |
| *stxbp1a* | 0 | 2.361369 | 0.895 | 0.155 | 0 | 2 |
| *rho* | 0 | 3.811814 | 1 | 0.999 | 0 | 3 |
| *rom1b* | 0 | 3.503928 | 0.839 | 0.44 | 0 | 3 |
| *si:ch211-113d22.2* | 0 | 3.366633 | 0.88 | 0.607 | 0 | 3 |
| *si:dkey-22i16.2* | 0 | 3.30342 | 0.795 | 0.314 | 0 | 3 |
| *cnga1* | 0 | 3.213976 | 0.773 | 0.27 | 0 | 3 |
| *gnat1* | 0 | 3.16765 | 0.989 | 0.937 | 0 | 3 |
| *rhol* | 0 | 2.969203 | 0.657 | 0.144 | 0 | 3 |
| *pde6g* | 9E-276 | 2.940326 | 0.91 | 0.758 | 1.7E-271 | 3 |
| *rom1a* | 0 | 2.721132 | 0.645 | 0.169 | 0 | 3 |
| *elovl4b* | 2E-299 | 2.710312 | 0.73 | 0.299 | 3.8E-295 | 3 |
| *dct* | 0 | 4.388483 | 0.937 | 0.144 | 0 | 4 |
| *lrp1aa* | 0 | 4.241001 | 0.837 | 0.125 | 0 | 4 |
| *pmela* | 0 | 4.231526 | 0.87 | 0.087 | 0 | 4 |
| *ambp-1* | 0 | 4.227394 | 0.883 | 0.124 | 0 | 4 |
| *rgrb* | 0 | 4.171871 | 0.99 | 0.453 | 0 | 4 |
| *cst3* | 0 | 3.888058 | 0.999 | 0.911 | 0 | 4 |
| *pttg1ipb* | 0 | 3.876994 | 0.904 | 0.208 | 0 | 4 |
| *tyrp1b* | 0 | 3.796407 | 0.896 | 0.132 | 0 | 4 |
| *mb* | 7.1E-138 | 3.639 | 0.484 | 0.189 | 1.3E-133 | 4 |
| *itih1* | 0 | 3.627927 | 0.701 | 0.058 | 0 | 4 |
| *slc6a1b* | 9.7E-165 | 2.822531 | 0.45 | 0.118 | 1.8E-160 | 5 |
| *slc6a1a* | 1.3E-203 | 2.753738 | 0.464 | 0.105 | 2.5E-199 | 5 |
| *slc6a9* | 3.28E-79 | 2.753095 | 0.297 | 0.094 | 6.2E-75 | 5 |
| *grin1a* | 1.6E-278 | 2.724345 | 0.751 | 0.234 | 3.1E-274 | 5 |
| *slc32a1* | 8.5E-289 | 2.692321 | 0.601 | 0.133 | 1.6E-284 | 5 |
| *syt2a* | 2.8E-175 | 2.672815 | 0.425 | 0.1 | 5.4E-171 | 5 |
| *sv2a* | 3.6E-282 | 2.600473 | 0.7 | 0.192 | 6.8E-278 | 5 |
| *syt1a* | 0 | 2.561834 | 0.804 | 0.221 | 0 | 5 |
| *LOC103908668* | 5.2E-152 | 2.318628 | 0.515 | 0.172 | 9.8E-148 | 5 |
| *gabrb2* | 9.4E-152 | 2.224089 | 0.403 | 0.102 | 1.8E-147 | 5 |
| *arr3b* | 0 | 4.137515 | 0.981 | 0.459 | 0 | 6 |
| *gngt2b* | 0 | 3.367986 | 1 | 0.971 | 0 | 6 |
| *es1* | 7.5E-308 | 3.227079 | 1 | 0.973 | 1.4E-303 | 6 |
| *zgc:73359* | 0 | 2.952109 | 1 | 0.984 | 0 | 6 |
| *gnb3b* | 0 | 2.918634 | 1 | 0.773 | 0 | 6 |
| *si:dkey-97a13.12* | 0 | 2.768871 | 0.993 | 0.537 | 0 | 6 |
| *gnat2* | 0 | 2.720752 | 0.998 | 0.825 | 0 | 6 |
| *si:dkey-126g1.9* | 0 | 2.703528 | 0.99 | 0.263 | 0 | 6 |
| *si:cabz01076231.1* | 0 | 2.525011 | 0.988 | 0.279 | 0 | 6 |
| *kera* | 0 | 2.510421 | 0.843 | 0.115 | 0 | 6 |
| *LOC103909376* | 2.1E-120 | 2.831975 | 0.389 | 0.088 | 4E-116 | 7 |
| *si:ch211-222l21.1* | 1.2E-265 | 2.615485 | 0.603 | 0.102 | 2.3E-261 | 7 |
| *cxxc5a* | 5.8E-266 | 2.249814 | 0.49 | 0.065 | 1.1E-261 | 7 |
| *sox4a* | 1.9E-181 | 2.189421 | 0.528 | 0.107 | 3.6E-177 | 7 |
| *tmsb* | 1.4E-263 | 2.146742 | 0.339 | 0.028 | 2.7E-259 | 7 |
| *rplp2l* | 2.5E-181 | 2.079537 | 0.954 | 0.62 | 4.7E-177 | 7 |
| *rpl27a* | 3.2E-157 | 2.048143 | 0.782 | 0.335 | 6E-153 | 7 |
| *eef1g* | 1.4E-157 | 1.9788 | 0.885 | 0.504 | 2.7E-153 | 7 |
| *fabp7a* | 7.9E-110 | 1.963597 | 0.472 | 0.129 | 1.5E-105 | 7 |
| *rps20* | 8.3E-184 | 1.961726 | 0.984 | 0.789 | 1.6E-179 | 7 |
| *mdka* | 0 | 6.630412 | 1 | 0.191 | 0 | 8 |
| *si:dkey-273o13.3* | 0 | 5.691251 | 0.981 | 0.011 | 0 | 8 |
| *rprmb* | 0 | 4.765208 | 0.956 | 0.018 | 0 | 8 |
| *aqp9a* | 0 | 4.698649 | 0.96 | 0.013 | 0 | 8 |
| *lbh* | 0 | 4.632958 | 0.907 | 0.034 | 0 | 8 |
| *atp1b1a* | 0 | 4.525271 | 0.996 | 0.153 | 0 | 8 |
| *ompa* | 0 | 4.482134 | 0.879 | 0.011 | 0 | 8 |
| *LOC101882145* | 0 | 4.450836 | 0.911 | 0.026 | 0 | 8 |
| *plekhd1* | 0 | 4.391484 | 0.939 | 0.047 | 0 | 8 |
| *slc4a5* | 0 | 4.372114 | 0.883 | 0.031 | 0 | 8 |
| *rbpms2a* | 0 | 4.935184 | 0.941 | 0.047 | 0 | 9 |
| *si:dkey-7j14.5* | 0 | 4.121109 | 0.941 | 0.093 | 0 | 9 |
| *rbpms2b* | 0 | 3.522256 | 0.836 | 0.038 | 0 | 9 |
| *wu:fj58g06-1* | 5.9E-150 | 3.466013 | 0.301 | 0.038 | 1.1E-145 | 9 |
| *nrgna* | 0 | 3.227394 | 0.738 | 0.116 | 0 | 9 |
| *cplx2l* | 0 | 3.207703 | 0.818 | 0.114 | 0 | 9 |
| *isl2b* | 0 | 3.078175 | 0.572 | 0.007 | 0 | 9 |
| *rab6ba* | 0 | 3.028613 | 0.815 | 0.05 | 0 | 9 |
| *adcyap1b* | 0 | 2.975482 | 0.469 | 0.005 | 0 | 9 |
| *inab* | 0 | 2.91146 | 0.711 | 0.014 | 0 | 9 |
| *ba1* | 2.6E-251 | 7.558887 | 1 | 0.593 | 4.9E-247 | 10 |
| *hbaa1* | 2.7E-242 | 7.446047 | 1 | 0.689 | 5.1E-238 | 10 |
| *ba1l* | 0 | 6.47716 | 1 | 0.123 | 0 | 10 |
| *si:ch211-5k11.8* | 0 | 6.421534 | 1 | 0.209 | 0 | 10 |
| *si:ch211-250g4.3* | 0 | 5.997521 | 0.978 | 0.008 | 0 | 10 |
| *creg1* | 0 | 5.112928 | 0.973 | 0.086 | 0 | 10 |
| *hbba2* | 0 | 4.301128 | 0.603 | 0.013 | 0 | 10 |
| *hbaa2* | 0 | 3.759552 | 0.427 | 0.011 | 0 | 10 |
| *mibp* | 0 | 3.053733 | 0.608 | 0.008 | 0 | 10 |
| *blvrb* | 0 | 2.790517 | 0.597 | 0.066 | 0 | 10 |
| *rpe65a* | 0 | 4.437179 | 0.99 | 0.163 | 0 | 11 |
| *rlbp1b* | 3.6E-280 | 4.27623 | 0.99 | 0.267 | 6.8E-276 | 11 |
| *thbs1b* | 0 | 3.950067 | 0.997 | 0.096 | 0 | 11 |
| *f3b* | 0 | 3.873123 | 0.926 | 0.057 | 0 | 11 |
| *rlbp1b-1* | 2.6E-281 | 3.780893 | 0.993 | 0.254 | 4.9E-277 | 11 |
| *pnp4a* | 0 | 3.724114 | 0.987 | 0.059 | 0 | 11 |
| *fabp11b* | 7.7E-270 | 3.695808 | 0.977 | 0.231 | 1.4E-265 | 11 |
| *rbp5* | 8.8E-228 | 3.687865 | 0.993 | 0.352 | 1.7E-223 | 11 |
| *dusp2* | 0 | 3.670689 | 0.997 | 0.203 | 0 | 11 |
| *ca9* | 0 | 3.629561 | 0.987 | 0.057 | 0 | 11 |
| *ptgdsb.2* | 0 | 6.069471 | 0.996 | 0.085 | 0 | 12 |
| *apoeb* | 0 | 5.747821 | 0.993 | 0.1 | 0 | 12 |
| *zgc:153704* | 0 | 5.616926 | 0.978 | 0.054 | 0 | 12 |
| *rlbp1a* | 0 | 5.247792 | 1 | 0.061 | 0 | 12 |
| *igfbp1a* | 0 | 5.134332 | 0.859 | 0.028 | 0 | 12 |
| *cebpd* | 0 | 4.965995 | 0.909 | 0.02 | 0 | 12 |
| *ptgdsb.1* | 0 | 4.844963 | 0.92 | 0.04 | 0 | 12 |
| *cahz* | 0 | 4.839412 | 0.993 | 0.119 | 0 | 12 |
| *icn* | 0 | 4.645766 | 0.986 | 0.053 | 0 | 12 |
| *zgc:195173* | 0 | 4.495464 | 0.957 | 0.082 | 0 | 12 |
| *slc18a3a* | 0 | 4.00923 | 0.829 | 0.019 | 0 | 13 |
| *mdkb* | 1.4E-194 | 3.845208 | 0.996 | 0.388 | 2.7E-190 | 13 |
| *sox2* | 0 | 3.819129 | 0.72 | 0.029 | 0 | 13 |
| *rnd3b* | 0 | 3.745552 | 0.463 | 0.015 | 0 | 13 |
| *kiaa0040* | 0 | 3.535853 | 0.78 | 0.038 | 0 | 13 |
| *rgs3a* | 2.1E-155 | 3.349586 | 0.813 | 0.219 | 4E-151 | 13 |
| *arl4ab* | 2.3E-244 | 3.303692 | 0.756 | 0.11 | 4.4E-240 | 13 |
| *syt1a* | 6E-224 | 3.09087 | 0.992 | 0.24 | 1.1E-219 | 13 |
| *slit2* | 5.3E-155 | 2.999045 | 0.455 | 0.056 | 1E-150 | 13 |
| *zgc:101840* | 3.6E-248 | 2.947038 | 0.679 | 0.082 | 6.7E-244 | 13 |
| *icn* | 0 | 4.772532 | 0.922 | 0.065 | 0 | 14 |
| *sepp1a-1* | 2E-186 | 4.273613 | 0.766 | 0.079 | 3.9E-182 | 14 |
| *rlbp1a* | 1.7E-274 | 4.048091 | 0.906 | 0.074 | 3.2E-270 | 14 |
| *sepp1a* | 1.8E-170 | 4.039914 | 0.664 | 0.063 | 3.4E-166 | 14 |
| *fxyd6l* | 4.2E-150 | 3.916246 | 0.906 | 0.154 | 8E-146 | 14 |
| *cahz* | 3E-162 | 3.776796 | 0.891 | 0.131 | 5.6E-158 | 14 |
| *zgc:153704* | 1.8E-205 | 3.651931 | 0.758 | 0.067 | 3.4E-201 | 14 |
| *cdo1* | 7E-176 | 3.524638 | 0.742 | 0.076 | 1.3E-171 | 14 |
| *LOC101885164* | 3.46E-66 | 3.308374 | 0.844 | 0.356 | 6.54E-62 | 14 |
| *s100a10b* | 3.6E-215 | 3.128883 | 0.742 | 0.06 | 6.9E-211 | 14 |
| *fabp7a* | 1.8E-162 | 4.383033 | 0.935 | 0.135 | 3.4E-158 | 15 |
| *her4.3* | 9.6E-222 | 4.014462 | 0.274 | 0.006 | 1.8E-217 | 15 |
| *her15.1* | 1.5E-184 | 3.997166 | 0.419 | 0.02 | 2.9E-180 | 15 |
| *hmgn2* | 3.45E-46 | 3.862143 | 0.903 | 0.425 | 6.51E-42 | 15 |
| *LOC100534909* | 5.2E-188 | 3.837915 | 0.419 | 0.02 | 9.9E-184 | 15 |
| *LOC100148329* | 2.9E-201 | 3.669825 | 0.298 | 0.009 | 5.5E-197 | 15 |
| *id1* | 1.8E-142 | 3.585239 | 0.54 | 0.046 | 3.4E-138 | 15 |
| *socs3a* | 2.57E-68 | 3.530901 | 0.508 | 0.08 | 4.85E-64 | 15 |
| *ggctb* | 7.67E-32 | 3.517551 | 0.298 | 0.053 | 1.45E-27 | 15 |
| *si:dkey-238o13.4* | 7.5E-169 | 3.238404 | 0.621 | 0.051 | 1.4E-164 | 15 |
| *s100a10b* | 2.7E-140 | 5.259996 | 0.625 | 0.062 | 5.2E-136 | 16 |
| *nrgna* | 3.9E-172 | 4.525266 | 0.946 | 0.131 | 7.3E-168 | 16 |
| *syt5b* | 8.9E-183 | 4.411684 | 0.982 | 0.135 | 1.7E-178 | 16 |
| *plk2a* | 1.3E-306 | 3.71305 | 0.438 | 0.011 | 2.5E-302 | 16 |
| *rdh10a* | 5.7E-158 | 3.668155 | 0.634 | 0.054 | 1.1E-153 | 16 |
| *LOC110438400* | 0 | 3.492214 | 0.723 | 0.034 | 0 | 16 |
| *dgkaa* | 0 | 3.35081 | 0.804 | 0.018 | 0 | 16 |
| *rdh10a-1* | 3.5E-132 | 3.077272 | 0.464 | 0.033 | 6.6E-128 | 16 |
| *plch2a* | 4.7E-169 | 3.055334 | 0.866 | 0.1 | 8.9E-165 | 16 |
| *uts1* | 1.11E-94 | 3.023464 | 0.348 | 0.026 | 2.09E-90 | 16 |
| *chgb* | 0 | 5.855756 | 0.962 | 0.042 | 0 | 17 |
| *agrn* | 1.28E-49 | 4.365505 | 0.387 | 0.06 | 2.42E-45 | 17 |
| *bhlhe22* | 4E-238 | 4.249286 | 0.981 | 0.091 | 7.6E-234 | 17 |
| *meis2b* | 2E-255 | 3.44151 | 0.943 | 0.073 | 3.8E-251 | 17 |
| *slc18a3a* | 0 | 3.414063 | 0.821 | 0.028 | 0 | 17 |
| *syt9b* | 0 | 3.390629 | 0.726 | 0.026 | 0 | 17 |
| *anos1a* | 1.1E-249 | 3.264021 | 0.915 | 0.068 | 2E-245 | 17 |
| *phactr3b* | 3.5E-127 | 3.123554 | 0.953 | 0.164 | 6.6E-123 | 17 |
| *kcnc3a* | 6.6E-307 | 3.04103 | 0.962 | 0.06 | 1.2E-302 | 17 |
| *zgc:194629* | 0 | 2.752403 | 0.396 | 0.005 | 0 | 17 |
| *cd59* | 0 | 7.808436 | 1 | 0.016 | 0 | 18 |
| *plp1b* | 0 | 7.33091 | 1 | 0.01 | 0 | 18 |
| *cldnk* | 0 | 7.209105 | 1 | 0.005 | 0 | 18 |
| *cd9b* | 0 | 6.571579 | 1 | 0.005 | 0 | 18 |
| *si:dkey-200l5.4* | 0 | 5.958872 | 0.989 | 0.004 | 0 | 18 |
| *mbpa* | 0 | 5.611453 | 0.989 | 0.01 | 0 | 18 |
| *tuba8l3* | 0 | 5.520353 | 0.989 | 0.005 | 0 | 18 |
| *si:rp71-19m20.1* | 4.6E-253 | 5.28379 | 0.989 | 0.073 | 8.6E-249 | 18 |
| *cd82a* | 0 | 5.214505 | 0.989 | 0.041 | 0 | 18 |
| *zwi* | 0 | 5.211844 | 0.989 | 0.008 | 0 | 18 |
| *apoc1* | 0 | 5.782885 | 0.884 | 0.029 | 0 | 19 |
| *pfn1* | 7.2E-195 | 5.074421 | 0.884 | 0.058 | 1.4E-190 | 19 |
| *cd74a* | 0 | 4.617333 | 0.754 | 0.013 | 0 | 19 |
| *LOC110439470* | 0 | 4.073807 | 0.609 | 0.01 | 0 | 19 |
| *lgals3bpb* | 0 | 3.818945 | 0.609 | 0.01 | 0 | 19 |
| *vmp1* | 2.21E-18 | 3.807155 | 0.377 | 0.092 | 4.18E-14 | 19 |
| *lgals2a* | 8E-247 | 3.522558 | 0.652 | 0.022 | 1.5E-242 | 19 |
| *fabp11a* | 1.1E-102 | 3.505988 | 0.406 | 0.021 | 2.08E-98 | 19 |
| *cd74b* | 0 | 3.487105 | 0.565 | 0.011 | 0 | 19 |
| *arpc1b* | 0 | 3.378654 | 0.71 | 0.009 | 0 | 19 |
| *apoc1* | 0 | 7.283479 | 0.985 | 0.029 | 0 | 20 |
| *cd74a* | 0 | 5.993695 | 1 | 0.012 | 0 | 20 |
| *lgals3bpb* | 0 | 5.980537 | 0.985 | 0.008 | 0 | 20 |
| *fabp11a* | 0 | 5.878762 | 0.785 | 0.019 | 0 | 20 |
| *zgc:92066* | 2.7E-54 | 5.303331 | 1 | 0.381 | 5.1E-50 | 20 |
| *cmklr1* | 0 | 5.14607 | 1 | 0.005 | 0 | 20 |
| *vmp1* | 4.3E-166 | 5.08591 | 1 | 0.088 | 8.1E-162 | 20 |
| *si:busm1-266f07.2* | 0 | 4.799624 | 0.969 | 0.013 | 0 | 20 |
| *pfn1* | 5E-237 | 4.793024 | 1 | 0.057 | 9.4E-233 | 20 |
| *cd74b* | 0 | 4.641042 | 0.985 | 0.009 | 0 | 20 |
| *si:ch211-214p16.1* | 0 | 6.635844 | 0.875 | 0.003 | 0 | 21 |
| *cxcr4b* | 0 | 5.404781 | 0.786 | 0.01 | 0 | 21 |
| *b2m* | 1.5E-104 | 5.155761 | 1 | 0.127 | 2.8E-100 | 21 |
| *ccr9a* | 0 | 4.939505 | 0.786 | 0.005 | 0 | 21 |
| *srgn-1* | 0 | 4.828549 | 0.929 | 0.015 | 0 | 21 |
| *pfn1* | 4.8E-165 | 4.485086 | 0.911 | 0.059 | 9.1E-161 | 21 |
| *LOC100151049* | 0 | 4.183341 | 0.857 | 0.011 | 0 | 21 |
| *si:dkey-27i16.2* | 9.8E-295 | 4.107388 | 0.714 | 0.018 | 1.8E-290 | 21 |
| *ucp2* | 7.2E-258 | 4.094345 | 0.857 | 0.031 | 1.4E-253 | 21 |
| *cebpb* | 1.4E-108 | 4.050617 | 0.696 | 0.05 | 2.6E-104 | 21 |
| *slc6a9* | 1.65E-77 | 6.020585 | 0.947 | 0.103 | 3.12E-73 | 22 |
| *tubb5* | 8E-165 | 5.812786 | 1 | 0.05 | 1.5E-160 | 22 |
| *chga* | 5.06E-98 | 5.139301 | 0.842 | 0.06 | 9.56E-94 | 22 |
| *si:dkey-238o13.4* | 3.3E-126 | 4.751125 | 0.921 | 0.055 | 6.2E-122 | 22 |
| *kidins220a* | 0 | 4.70627 | 0.947 | 0.003 | 0 | 22 |
| *kcnip1b-1* | 9.7E-150 | 4.493498 | 0.842 | 0.038 | 1.8E-145 | 22 |
| *kcnd1* | 1.5E-124 | 4.486649 | 0.868 | 0.049 | 2.8E-120 | 22 |
| *anos1a* | 8.2E-98 | 4.18251 | 0.921 | 0.073 | 1.55E-93 | 22 |
| *rasd1* | 1.06E-77 | 4.164931 | 0.711 | 0.052 | 1.99E-73 | 22 |
| *tbx3a* | 1.4E-130 | 4.120598 | 0.947 | 0.056 | 2.6E-126 | 22 |

Table S3. **Number and relative proportion of selected cell types in two WT and P23H datasets**

|  | **V2 run** | | | | **V3 run** | | | |
| --- | --- | --- | --- | --- | --- | --- | --- | --- |
|  | **WT** | | **P23H** | | **WT** | | **P23H** | |
|  | # | % | # | % | # | % | # | % |
| Rods | 500 | 13.1 | 211 | 4.7 | 869 | 6.4 | 501 | 3.2 |
| Cones | 311 | 8.1 | 426 | 9.5 | 1759 | 13.0 | 1138 | 7.3 |
| New rods | 56 | 1.5 | 631 | 14.1 | 150 | 1.1 | 583 | 3.8 |
| RPC | 89 | 2.3 | 442 | 9.9 | 62 | 0.5 | 341 | 2.2 |
| RPE | 71 | 1.9 | 32 | 0.7 | 1080 | 8.0 | 156 | 1.0 |

Table S4. **Parameters of V3 datasets**

| Sample | Cells Submitted to Make Library | Total Read Number | Median Reads Per Cell | Reported Cells | Total Genes detected | Reported Median Genes | Filter Parameters | Cells Left After Filter | Median nGenes after filter |
| --- | --- | --- | --- | --- | --- | --- | --- | --- | --- |
| P23H | 14,000-24,000 | 608,597,236 | 39,050 | 15,585 | 26,375 | 684 | 200>x<4700 | 15,511 | 684 |
| WT | 14,000-24,000 | 967,526,548 | 69,277 | 13,966 | 25,673 | 658 | 200>x<6000 | 13,552 | 669 |

Table S5. **Rod bipolar cell protein-protein interaction networks inferred by STRING analysis**

| **WT rod bipolar cell reactome pathways** | | | | |
| --- | --- | --- | --- | --- |
| **Pathway** | **Description** | **Count in**  **Network** | **Strength** | **False Discovery Rate** |
| DRE390471 | Association of TriC/CCt with target proteins during biosynthesis | 6/9 | 1.81 | 2.80e-06 |
| DRE-8866427 | VLDRLR internalization and degradation | 3/11 | 1.42 | 0.0071 |
| DRE-111447 | Activation of BAD and translocation to mitochondria | 2/9 | 1.33 | 0.0280 |
| DRE-1445148 | Translocation of SLC2A4 (GLUT4) to the plasma membrane | 2/10 | 1.29 | 0.0312 |
| DRE-3371497 | HSP90 chaperone cycle for steroid hormone receptors (SHR) | 4/21 | 1.27 | 0.0043 |

| **P23H rod bipolar cell reactome pathways** | | | | |
| --- | --- | --- | --- | --- |
| **Pathway** | **Description** | **Count in**  **Network** | **Strength** | **False Discovery Rate** |
| DRE-8949613 | Cristae formation | 4/20 | 1.31 | 0.0046 |
| DRE-163210 | Formation of ATP by chemiosmotic coupling | 4/20 | 1.31 | 0.0046 |
| DRE-1592230 | Mitochondrial biogenesis | 5/32 | 1.2 | 0.0026 |
| DRE-77387 | Insulin receptor recycling | 4/29 | 1.15 | 0.0102 |
| DRE-1222556 | ROS, RNS production in phagocytes | 4/31 | 1.12 | 0.0119 |

**References (Table S1)**

1. Di Donato, V., et al., *Characterization of the calcium binding protein family in zebrafish.* PLoS One, 2013. **8**(1): p. e53299.

2. Jin, K., et al., *Tfap2a and 2b act downstream of Ptf1a to promote amacrine cell differentiation during retinogenesis.* Mol Brain, 2015. **8**: p. 28.

3. Puthussery, T., J. Gayet-Primo, and W.R. Taylor, *Localization of the calcium-binding protein secretagogin in cone bipolar cells of the mammalian retina.* J Comp Neurol, 2010. **518**(4): p. 513-25.

4. Yan, W., et al., *Mouse Retinal Cell Atlas: Molecular Identification of over Sixty Amacrine Cell Types.* J Neurosci, 2020. **40**(27): p. 5177-5195.

5. Woods, S.M., et al., *A comparative analysis of rod bipolar cell transcriptomes identifies novel genes implicated in night vision.* Sci Rep, 2018. **8**(1): p. 5506.

6. Vitorino, M., et al., *Vsx2 in the zebrafish retina: restricted lineages through derepression.* Neural Dev, 2009. **4**: p. 14.

7. Kim, D.S., et al., *Identification of molecular markers of bipolar cells in the murine retina.* J Comp Neurol, 2008. **507**(5): p. 1795-810.

8. Sun, C., C. Galicia, and D.L. Stenkamp, *Transcripts within rod photoreceptors of the Zebrafish retina.* BMC Genomics, 2018. **19**(1): p. 127.

9. Clarke, G., et al., *Rom-1 is required for rod photoreceptor viability and the regulation of disk morphogenesis.* Nat Genet, 2000. **25**(1): p. 67-73.

10. Lagman, D., et al., *Transducin duplicates in the zebrafish retina and pineal complex: differential specialisation after the teleost tetraploidisation.* PLoS One, 2015. **10**(3): p. e0121330.

11. Stearns, G., et al., *A mutation in the cone-specific pde6 gene causes rapid cone photoreceptor degeneration in zebrafish.* J Neurosci, 2007. **27**(50): p. 13866-74.

12. Zhang, Q., et al., *Comparative analysis and expression of CLUL1, a cone photoreceptor-specific gene.* Invest Ophthalmol Vis Sci, 2003. **44**(10): p. 4542-9.

13. Klaassen, L.J., et al., *Specific connectivity between photoreceptors and horizontal cells in the zebrafish retina.* J Neurophysiol, 2016. **116**(6): p. 2799-2814.

14. Wilson, S.G., et al., *Tracking the fate of her4 expressing cells in the regenerating retina using her4:Kaede zebrafish.* Exp Eye Res, 2016. **145**: p. 75-87.

15. Ramachandran, R., X.F. Zhao, and D. Goldman, *Insm1a-mediated gene repression is essential for the formation and differentiation of Muller glia-derived progenitors in the injured retina.* Nat Cell Biol, 2012. **14**(10): p. 1013-23.

16. Cheng, Y.C., et al., *The transcription factor hairy/E(spl)-related 2 induces proliferation of neural progenitors and regulates neurogenesis and gliogenesis.* Dev Biol, 2015. **397**(1): p. 116-28.

17. Zhang, L., et al., *Expression profiling of the RPE in zebrafish smarca4 mutant revealed altered signals that potentially affect RPE and retinal differentiation.* Mol Vis, 2014. **20**: p. 56-72.

18. Plaza Reyes, A., et al., *Identification of cell surface markers and establishment of monolayer differentiation to retinal pigment epithelial cells.* Nat Commun, 2020. **11**(1): p. 1609.

19. Rodriguez, A.R., L.P. de Sevilla Muller, and N.C. Brecha, *The RNA binding protein RBPMS is a selective marker of ganglion cells in the mammalian retina.* J Comp Neurol, 2014. **522**(6): p. 1411-43.

20. Muzyka, V.V. and T.C. Badea, *Genetic interplay between transcription factor Pou4f1/Brn3a and neurotrophin receptor Ret in retinal ganglion cell type specification.* Neural Dev, 2021. **16**(1): p. 5.

21. Kolsch, Y., et al., *Molecular classification of zebrafish retinal ganglion cells links genes to cell types to behavior.* Neuron, 2021. **109**(4): p. 645-662 e9.

22. Takada, N. and B. Appel, *Identification of genes expressed by zebrafish oligodendrocytes using a differential microarray screen.* Dev Dyn, 2010. **239**(7): p. 2041-7.

23. Wiltbank, A.T., et al., *Cd59 and inflammation regulate Schwann cell development.* Elife, 2022. **11**.

24. Nakamura, Y., R. Iwamoto, and E. Mekada, *Expression and distribution of CD9 in myelin of the central and peripheral nervous systems.* Am J Pathol, 1996. **149**(2): p. 575-83.

25. Li, W.H., et al., *Zebrafish Lbh-like Is Required for Otx2-mediated Photoreceptor Differentiation.* Int J Biol Sci, 2015. **11**(6): p. 688-700.

26. Thummel, R., et al., *Characterization of Muller glia and neuronal progenitors during adult zebrafish retinal regeneration.* Exp Eye Res, 2008. **87**(5): p. 433-44.

27. Sifuentes, C.J., et al., *Rapid, Dynamic Activation of Muller Glial Stem Cell Responses in Zebrafish.* Invest Ophthalmol Vis Sci, 2016. **57**(13): p. 5148-5160.

28. Mitchell, D.M., et al., *Regeneration associated transcriptional signature of retinal microglia and macrophages.* Sci Rep, 2019. **9**(1): p. 4768.
